# Impact of combined Hydrus Microstent implantation and cataract surgery on diurnal intraocular pressure range: a longitudinal, retrospective study

**DOI:** 10.1186/s12886-026-05247-0

**Published:** 2026-08-29

**Authors:** Patrick Thelen, Thomas Reinhard, Gloria Helmers, Daniel Böhringer, Jan Lübke, Philip Keye

**Affiliations:** Eye Center, Medical Center – University of Freiburg, Faculty of Medicine, University of Freiburg, Freiburg, Germany

**Keywords:** Hydrus Microstent, Diurnal IOP range, Glaucoma surgery

## Abstract

**Purpose:**

To evaluate the impact of combined Hydrus Microstent implantation and cataract surgery on the 24-hour intraocular pressure (IOP) range in patients with open-angle glaucoma.

**Patients and methods:**

This retrospective, single-center study included 125 consecutive eyes of 125 patients who underwent combined Hydrus Microstent implantation and cataract surgery. Inpatient 24-hour IOP profiles (comprising daytime Goldmann applanation tonometry and nocturnal iCare rebound tonometry) from before and after surgery were analyzed. Surgical success was defined as a postoperative mean 24-hour IOP ≤ 21 mmHg with either a ≥ 20% reduction from baseline or a reduction in glaucoma medication burden. The primary outcome measures were changes in mean 24-hour IOP, maximum 24-hour IOP, and 24-hour IOP range (defined as the difference between the maximal and minimal 24-hour IOP).

**Results:**

Mean preoperative 24-hour IOP of 21.0 ± 5.5 mmHg decreased to 16.5 ± 4.0 mmHg postoperatively (mean reduction: 4.5 mmHg) at a mean follow-up of 76 days (range 7–338 days; median 61 days; IQR 35–91). The 24-hour IOP range significantly decreased from 10.7 ± 6.5 mmHg to 7.7 ± 4.0 mmHg (*p* < 0.001), and mean maximum 24-hour IOP decreased by 6.2 ± 5.4 mmHg. The number of glaucoma medications was reduced from 2.4 ± 1.2 to 0.6 ± 1.1, with 74.4% of patients becoming medication-free. Overall surgical success was achieved in 88.0% of patients.

**Conclusion:**

Combined Hydrus Microstent implantation and cataract surgery significantly reduced glaucoma medication burden and effectively decreased 24-hour IOP range and pressure spikes.

**Précis:**

Combined Hydrus Microstent implantation with cataract surgery reduced diurnal intraocular pressure range and pressure peaks while lowering mean intraocular pressure, indicating improved pressure stability beyond average reduction alone.

**Supplementary Information:**

The online version contains supplementary material available at https://doi.org/10.1186/s12886-026-05247-0.

## Introduction

Primary open-angle glaucoma (POAG) is a chronic, progressive optic neuropathy characterized by damage to the optic nerve and corresponding visual field loss [1, 2]. It is the most common form of glaucoma and a leading cause of irreversible blindness worldwide [2, 3]. POAG is often associated with elevated intraocular pressure (IOP), which is considered a significant risk factor, although the condition can also occur with normal IOP levels [1, 3]. While lowering mean IOP is the primary goal of therapy, recent evidence suggests that excessive IOP range and IOP peaks may be independent risk factors for visual field progression in OAG [4, 5]. Large IOP range may exert mechanical stress on the lamina cribrosa, potentially accelerating glaucomatous damage even when IOP appears controlled during clinic visits. Effective management strategies aim to reduce IOP and slow disease progression, preserving vision and improving patients’ quality of life [6]. Reduction of IOP can be achieved with topical medications, laser procedures such as selective laser trabeculoplasty (SLT), either as a first-line therapy or following prior glaucoma interventions [7, 8], or incisional glaucoma surgery. In cases of progressive or advanced glaucoma, filtrating surgical procedures may be required, while angle procedures may be a viable option in cases of early and moderate glaucoma [2, 9, 10]. In recent years, various minimally invasive glaucoma surgery (MIGS) procedures have emerged, mainly as a viable adjunct to cataract surgery, providing an opportunity to address both cataract-related vision loss and glaucoma-related IOP management in a single surgical intervention [11–13].

The Hydrus Microstent (Alcon AG, Freiburg, Switzerland), a crescent-shaped, flexible device, is designed to enhance aqueous humor outflow through Schlemm’s canal, making it an option for patients with mild to moderate POAG. The approximately 8 mm long stent is inserted into the Schlemm’s canal, opening it over about 90° and thereby improving the outflow of aqueous humor. Large-scale randomized controlled trials, notably the HORIZON study, have established the long-term efficacy and safety of the Hydrus Microstent combined with phacoemulsification, demonstrating sustained intraocular pressure reduction, decreased medication burden, and a significantly lower incidence of subsequent filtration surgeries compared to cataract surgery alone [11, 14–17].

This study reports the outcomes of Hydrus Microstent implantation combined with cataract surgery at a tertiary referral center. Unlike standard outpatient assessments, this analysis utilizes 24-hour inpatient IOP profiles to specifically address the procedure’s impact on IOP range and IOP peaks.

## Patients and methods

This retrospective monocentric analysis included patients who underwent combined Hydrus Microstent implantation and cataract surgery at a large university eye hospital. We identified eligible subjects via OPS Codes (5-131.61 for Hydrus Microstent; 5-144.5a for cataract surgery). The OPS is the official German classification for surgical procedures, ensuring a standardized documentation and forming a basis for managing healthcare resources within the national G-DRG framework (https://www.bfarm.de/EN/Code-systems/Classifications/OPS-ICHI/OPS/_node.html). Formal inclusion criteria were defined as: [1] combined phacoemulsification and Hydrus implantation, and [2] the availability of 24-hour IOP profiles for both the study eye and the fellow eye, both before and after surgery.

Regarding IOP measurements, two different devices were utilized: Goldmann Applanation Tonometry (GAT) was used for all daytime measurements with the patient in a sitting position, while iCare rebound tonometry was used for nocturnal measurements. For these, patients remained in a supine position to capture the physiological IOP state during sleep. In accordance with routine clinical practice, GAT measurements during the hospital stay were performed by different ophthalmologists, with a single measurement per timepoint. By default, the iCare device takes six single measurements to calculate IOP. While the use of two devices introduces a potential systematic bias, this protocol was chosen to balance the “gold standard” accuracy of GAT with the practical necessity of supine measurements at night. As part of the standard inpatient protocol for glaucoma patients at our institution, 24-hour pressure profiles were performed the day before surgery and included measurements at 12:00, 16:00, 20:00, 00:00 and 07:00. Postoperative 24-hour IOP profiles for the study eye were obtained either [1] during the inpatient stay for surgery of the fellow eye [2], during the inpatient stay for follow-up surgery of the study eye, or [3] during an inpatient stay for other reasons without any surgical intervention. The term “IOP range” used throughout this manuscript was defined as the difference between the maximal and minimal IOP recorded within a 24-hour profile. The time interval between surgery of the study eye and the follow-up IOP profile ranged from 7 to 338 days.

Clinical Efficacy was evaluated based on the analysis of IOP profiles. Specifically, the reduction in mean and maximum 24-hour IOP and the change in IOP range were chosen as clinical outcome parameters. Additional outcome parameters included the reduction in the number of glaucoma medications and a composite success criterion, defined as a postoperative IOP ≤ 21 mmHg combined with either an IOP reduction of ≥ 20% or a decrease in medication burden.

This study was conducted in accordance with the principles of the Declaration of Helsinki. Approval was obtained from the Ethics Committee of the University of Freiburg (Approval No. 24-1544-S1-retro). Statistical was performed using the open-source statistical program R (R version 4.4.3 (2025-02-28)). Descriptive statistics are reported as mean ± standard deviation (SD) or median with quartiles. Shapiro-Wilk test was used to test for normality and Wilcoxon signed-rank test was used to test for statistical significance. We performed a regression analysis to determine if the duration of follow-up or subtype of glaucoma had an impact on any of the IOP outcome parameters. A p-value ≤ 0.05 was considered statistically significant.

## Results

A total of 125 eyes from 125 patients were included. The baseline demographic and clinical characteristics are summarized in Table 1. The mean age at the time of surgery was 76.3 years and the cohort was evenly split between males (46.4%) and females (53.6%). The most prevalent glaucoma diagnosis was primary open-angle glaucoma (POAG) (67.2%), followed by pseudoexfoliative glaucoma (PXG) (23.2%). At baseline, the mean preoperative IOP was 21.0 (± 5.6) mmHg, and the mean number of glaucoma medications was 2.4 (± 1.2). The mean time between the two inpatient stays was 76 days (± 58.2) with a median of 61 days (IQR 35–91).

**Table 1:** Baseline characteristics

Number of patient eyes (*n*)	125
Mean Age at Surgery (in years)^1^	76.3 (± 7.88)
**Sex distribution²**	
Male	58 (46.4%)
Female	67 (53.6%)
**Glaucoma Diagnosis** ^**2**^	
Closed angle glaucoma (CAG)	2 (1.6%)
Normal-Tension glaucoma (NTG)	6 (4.8%)
Pigmentary glaucoma (PDG)	4 (3.2%)
Pseudoexfoliative glaucoma (PXG)	29 (23.2%)
Primary Open-Angle glaucoma (POAG)	84 (67.2%)
**Baseline IOP** ^**1**^	
Mean 24-hour IOP (in mmHg)	21.0 (± 5.6)
Maximum 24-hour IOP (in mmHg)	26.6 (± 8.0)
24-hour IOP range (in mmHg)	10.7 (± 6.5)
**Glaucoma therapy**	
Number of glaucoma medications^1^	2.4 (± 1.2)
Number of previous glaucoma surgeries or interventions²	
None	105 (84.0%)
1	17 (13.6%)
2	3 (2.4%)

*¹ Mean (± SD); ² n (%)*

IOP = Intraocular Pressure, glaucoma medications = Prostaglandin Analogues, Beta-Adrenergic Blockers, Alpha-2 Adrenergic Agonists, Carbonic Anhydrase Inhibitors (Topical), Cholinergic Agents (Miotics), Rho Kinase Inhibitors, Carbonic Anhydrase Inhibitors (Oral)

### Number of previous glaucoma surgeries or interventions

Most patients (79.9%) had no history of prior glaucoma surgery or laser intervention before undergoing the current procedure. Twenty-eight patients (18.2%) had undergone one previous glaucoma surgery or intervention (selective laser trabeculoplasty (SLT); *n* = 14, trabeculectomy (TE); *n* = 1, trabeculotomy; *n* = 1, transscleral cyclophotocoagulation (TSCPC); *n* = 1). Only three patients (1.9%) had a history of two prior interventions. The combinations included SLT and Preserflo^®^ MicroShunt, SLT followed by TE and SLT followed by TSCPC.

### Intraocular pressure

The mean 24-hour IOP decreased from a baseline of 21.0 ± 5.6 mmHg to 16.5 ± 3.9 mmHg, representing a mean reduction of 4.5 mmHg, or 21.4% from baseline. The mean maximum IOP recorded during the 24-hour profiles decreased by 6.2 ± 8.0 mmHg (23,3%) postoperatively. A reduction in mean 24-hour IOP was observed in 81.6% (102/125) of patients, while 84.8% (106/125) achieved a reduction or stabilization of their maximum IOP. Mean 24-hour IOP range significantly decreased by 28% from 10.7 ± 6.5 mmHg before surgery to 7.7 ± 4.0 mmHg postoperatively. All IOP outcome parameters are summarized in Table 2.

We performed a regression analysis to determine whether the duration of follow-up or the subtype of glaucoma had a significant impact on the IOP outcome parameters. Based on this analysis, the duration of follow-up did not show a statistically significant effect on any of the IOP outcome parameters. Regarding glaucoma subtype, patients with PXG showed a larger reduction of mean 24-hour IOP and maximum 24-hour IOP compared to patients with POAG. This result was statistically significant (*p* < 0.05). The results of the regression analysis are summarized in supplementary Table 1.

### Glaucoma medications

The mean number of glaucoma medications was reduced from 2.4 (± 1.2) at baseline to 0.6 (± 1.1) postoperatively (see Table 2). 74.4% (93/125) of patients were medication-free at the time of follow-up. A reduction in the number of medications was recorded in 83.2% (104/125) of treated eyes. Based on the composite success criterion (defined as a postoperative mean IOP ≤ 21 mmHg combined with either a ≥ 20% reduction of mean IOP or a reduction in medication) the overall success rate was 88.0% (110/125 patients).

**Table 2:** Results

	Pre-operative	Postoperative	*P value*
Mean 24-hour IOP (in mmHg)^1^	21.0 (± 5.6)	16.5 (± 3.9)	*P* < 0.001
Maximum 24-hour IOP (in mmHg)^1^	26.6 (± 8.0)	20.4 (± 5.4)	*P* < 0.001
24-hour IOP range (in mmHg)^1^	10.7 (± 6.5)	7.7 (± 4.0)	*P* < 0.001
Number of glaucoma medications^1^	2.4 (± 1.2)	0.6 (± 1.1)	*P* < 0.001

¹ Mean (± SD)

IOP = Intraocular Pressure, glaucoma medications = Prostaglandin Analogues, Beta-Adrenergic Blockers, Alpha-2 Adrenergic Agonists, Carbonic Anhydrase Inhibitors (Topical), Cholinergic Agents (Miotics), Rho Kinase Inhibitors, Carbonic Anhydrase Inhibitors (Oral)

### Safety

No severe intraoperative or sight-threatening postoperative complications, such as endophthalmitis, choroidal hemorrhage or persistent hypotony were recorded in the patient charts reviewed for this study.

11 of our 125 patients showed some sort of complication: Hyphema was seen in 3 of 125 eyes and postoperative IOP decompensation (defined as IOP > 26 mmHg) was seen in 8 of 125 eyes. All of these complications were transient and could be managed conservatively.

## Discussion

This retrospective analysis evaluates the real-world efficacy of the Hydrus Microstent combined with cataract surgery, incorporating 24-hour inpatient IOP profiles to assess outcome parameters beyond single measurements. Our data suggest that this combined procedure leads to a statistically significant reduction in mean IOP and a reduced number of glaucoma medications required. Furthermore, it mitigates IOP peaks and reduces IOP variability.

The most distinct and innovative finding of this study is the marked reduction in 24-hour IOP range, which decreased from 10.7 ± 6.5 mmHg to 7.7 ± 4.0 mmHg. This was accompanied by a mean decrease of 6.2 mmHg in maximum 24-hour IOP. While reduced IOP remains the standard benchmark for surgical success, it does not capture the full picture of IOP dynamics. Large range and intermittent IOP spikes are discussed as independent risk factors for visual field loss and glaucomatous progression [4, 5, 18]. By effectively mitigating peaks and decreasing the amplitude of IOP range, the procedure may offer a benefit that extends beyond what is reflected by mean IOP reduction alone. Unlike pivotal trials such as the HORIZON [11, 19] or the GEMINI study [4], which relied primarily on daytime office measurements, the use of 24-hour inpatient IOP profiles captures nocturnal and early-morning spikes often missed in outpatient settings. Regarding the 24-hour IOP profiles, three IOP values were obtained with GAT in a sitting position and two values were obtained with the iCare rebound tonometer. Generally, the agreement between GAT and iCare rebound tonometry measurements is reported to be satisfying with a trend to a clinically irrelevant underestimation of IOP in the iCare measurements [20]. Notably, due to measurements in supine body posture and the time of iCare measurements, IOP peaks may have occurred predominantly with this device [21]. Since the protocol was the same for pre- and postoperative IOP profiles, this should not introduce systematic bias.

Our study does not include a control group of eyes that received cataract surgery alone. However, the effect of cataract surgery on IOP in glaucomatous eyes has been well documented in several studies. In the Ocular Hypertension Treatment Study (OHTS), IOP after cataract surgery decreased from 19.8 ± 3.2 mmHg to 23.9 ± 3.2 mmHg (average decrease of 16.5%) [22]. Data from Poley et al. suggest the IOP reduction of cataract surgery to be proportional to preoperative IOP, with a more pronounced reduction in eyes with higher preoperative IOP values [23].

Data on the impact of glaucoma surgery on IOP variability primarily derive from studies on filtration surgery. Trabeculectomy has consistently been shown to reduce diurnal or circadian IOP range [24, 25]. While cataract surgery alone generally lowers mean IOP, it has been reported to have no effect on diurnal IOP variability in non-glaucomatous eyes and eyes with open-angle glaucoma [26]. In primary angle-closure glaucoma on the other hand, cataract surgery may reduce nocturnal or diurnal IOP range, likely due to anatomical widening of the anterior chamber angle [27, 28]. Since the present study virtually only included eyes with primary or secondary open-angle glaucoma (98.4% of eyes), it is reasonable to assume that the effect of combined Hydrus Microstent implantation and cataract surgery is largely attributable to the Hydrus Microstent.

Regarding other established outcome parameters, our findings align closely with published date, demonstrating a mean IOP reduction of 4.5 mmHg in a real-world population under topical glaucoma therapy. Interestingly, the regression analysis showed that patients with PXG benefitted more from the procedure than patients with POAG. This is in line with studies that showed a better outcome of different pressure-lowering surgical procedures in patients with PXG than in POAG patients [29]. A robust composite success criterion defined as a postoperative IOP ≤ 21 mmHg combined with either a pressure reduction of ≥ 20% or a decrease in medication burden was met in 88.0% of our cohort. This high rate of success mirrors the long-term outcomes reported by Ahmed et al. and real-world data from Laspas et al. [11, 14]. Regarding medication burden, the average number of active agents dropped from 2.4 to 0.6, allowing nearly three-quarters (74.4%) of patients to fully discontinue medical glaucoma therapy. Transitioning patients from complex polypharmacy to surgical management effectively eliminates the adherence barriers and ocular surface toxicity inherent to chronic drop regimens [30–32].

The safety profile observed in this series was excellent, with no sight-threatening complications. Transient hyphema and self-limiting IOP spikes were the only noted adverse events, consistent with the device’s established safety record [11, 16].

## Limitations

This study has some limitations, mostly inherent to its retrospective design. First, our study lacks a control group of eyes that received cataract surgery alone. As cataract surgery in general is mostly performed as an outpatient procedure, we were unable to provide a control group with 24-hour IOP profiles available. It is reasonable to assume that the IOP lowering effect of cataract surgery is also active in our study population. However, Kim et al. reported no statistically significant effect of cataract surgery alone on IOP fluctuation in non-glaucomatous eyes and eyes with open-angle glaucoma [26]. While a control group within our study would have been more robust, we refer to this published data regarding the effect of cataract surgery alone. The short duration and variability of follow-up in our study is another limitation. In order to reduce bias, we performed a regression analysis that suggested the effect on 24-hour IOP range to be independent from the duration of follow-up. However, the short follow-up period precludes definitive conclusions regarding the long-term efficacy of this combined surgical approach. Furthermore, patients with suboptimal outcomes in their first eye may not have been eligible for inclusion, as they may not have proceeded to surgery of the fellow eye or another inpatient stay with a 24-hour IOP profile. Regarding the IOP profiles, the use of iCare for nocturnal measurements in the supine position versus GAT for daytime measurements in the sitting position reflects a pragmatic approach to 24-hour IOP monitoring. While GAT and iCare measurements may not be perfectly interchangeable, our study focused on the intra-individual change in the 24-hour profile. By maintaining the same protocol for both the preoperative and postoperative IOP profiles, we ensured that the observed changes in IOP outcome parameters were reflective of the surgical intervention rather than being device-related. Lastly, in this study patients were not stratified for glaucoma severity in terms of visual field indices and patient at all stages of glaucoma were included. While this makes the results of the study well applicable to real-world glaucoma cohorts, it remains unclear if any subgroups profit above average.

## Conclusion

Hydrus Microstent implantation combined with cataract surgery is an effective treatment for open-angle glaucoma. Beyond simply lowering mean IOP and reducing medication dependence, the procedure reduces 24-hour IOP range and mitigates 24-hour IOP spikes. Given the evidence linking IOP variability to visual field progression, this stabilization might represent a therapeutic advantage of the device in the management of coexisting cataract and glaucoma.

## Supplementary Information

Below is the link to the electronic supplementary material.


Supplementary Material 1


## Data Availability

The data is available from the corresponding author on reasonable request.
