# Supplementary material for: Impact of combined Hydrus Microstent implantation and cataract surgery on diurnal intraocular pressure range: a longitudinal, retrospective study
Source: BMC Ophthalmol. 2026 Aug 29;26:530. doi: 10.1186/s12886-026-05247-0 (PMC13527981; doi:10.1186/s12886-026-05247-0)
Supplement: Supplementary file 1 [file 12886_2026_5247_MOESM1_ESM.docx]

|  | Estimate [95%-CI] | p value |
| --- | --- | --- |
| Intercept | 3.330839543 [0.98526542, 5.6764137] | 0.005762902* |
| Time_pre_post | -0.007931167 [-0.03026664, 0.0144043] | 0.483354661 |
| CAG | 3.283825913 [-7.05724001, 13.6248918] | 0.530695173 |
| NTG | -0.176518252 [-6.28512954, 5.9320930] | 0.954467342 |
| PG | -4.974105255 [-12.37093116, 2.4227206] | 0.185553952 |
| PXG | 1.994449366 [-1.12166261, 5.1105613] | 0.207504135 |

**Mean reduction of 24-hour IOP range**

|  | Estimate [95%-CI] | p value |
| --- | --- | --- |
| Intercept | 4.52800015 [2.92291945, 6.13308085] | 1.494303e-07* |
| Time_pre_post | -0.00511261 [-0.02039681, 0.01017159] | 5.090282e-01 |
| CAG | -2.64010618 [-9.71651669 4.43630433] | 4.615155e-01 |
| NTG | -2.01454312 [-6.19467711, 2.16559086] | 3.418790e-01 |
| PG | -0.22438545 [-5.28604708, 4.83727617] | 9.302002e-01 |
| PXG | 2.39763188 [0.26527070, 4.52999306] | 2.786775e-02* |

**Mean reduction of mean 24-hour IOP**

|  | Estimate [95%-CI] | p value |
| --- | --- | --- |
| Intercept | 6.69441786 [4.14912332, 9.23971239] | 8.106802e-07* |
| Time_pre_post | -0.01383325 [-0.03807053, 0.01040404] | 2.606955e-01 |
| CAG | -2.62234125 [-13.84392591, 8.59924341] | 6.444072e-01 |
| NTG | -2.05317502 [-8.68192096, 4.57557093] | 5.408403e-01 |
| PG | -2.88617450 [-10.91282366, 5.14047466] | 4.778630e-01 |
| PXG | 3.46635755 [0.08491558, 6.84779952] | 4.460450e-02* |

**Mean reduction of mean maximum IOP**
